# Rheumatism and Gout

**Published:** 1890-08

**Authors:** 


RHEUMATISM AND GOUT. By F. Leroy Satterlee, M. D., Professor of Chemistry, Medicine and Therapeutics in New York College of Dentistry.

This pamphlet of nearly one hundred pages, in giving the author's personal, as well as professional experience, with these maladies, presents very interesting and instructive details. From the labyrinth of mere theories he has held prominently up the more tenable hypotheses of pathology and treatment, and formulated valuable therapeutical resources that cannot fail to prove of service to those having to contend with these diseases. ROBT. W. W.

